# CAR-T therapy followed by allogeneic hematopoietic stem cell transplantation for refractory/relapsed acute B lymphocytic leukemia: Long-term follow-up results

**DOI:** 10.3389/fonc.2022.1048296

**Published:** 2023-01-04

**Authors:** Zhihui Li, Keyan Yang, Yanzhi Song, Yongqiang Zhao, Fan Wu, Xiaopei Wen, Jing Li, Xianxuan Wang, Teng Xu, Xiaoyu Zheng, Qinglong Zheng, Tong Wu

**Affiliations:** ^1^ Department of Bone Marrow Transplantation, Beijing Boren Hospital, Beijing, China; ^2^ Laboratory of Molecular Diagnostics, Beijing Boren Hospital, Beijing, China

**Keywords:** CAR-T therapy, B-ALL, allo-HSCT, somatic *TP53*, germline *EP300*

## Abstract

**Background:**

Patients with refractory/relapsed (r/r) acute B lymphocytic leukemia (B-ALL) can achieve complete response (CR) after chimeric antigen receptor T-cell (CAR-T) therapy, but recurrence occurs in the short term. To reduce recurrence and improve survival, CAR-T therapy followed by transplantation is a feasible option. We analyzed the long-term follow-up outcomes and the risk factors for allogeneic hematopoietic stem cell transplantation (allo-HSCT) after CR by CAR-T therapy in this study.

**Methods:**

A total of 144 patients who underwent allo-HSCT after CAR-T therapy in our hospital were enrolled in this study. Target gene analysis was performed in 137 r/r B-ALL patients receiving allo-HSCT after CR by CAR-T therapy. Among the 137 patients, 87 were evaluated for germline predisposition gene mutations, and 92 were evaluated for tumor somatic gene mutations using NGS. The clinical factors, germline predisposition gene and somatic gene mutations associated with the prognosis of patients receiving transplantation after CAR-T therapy were analyzed using univariate Cox regression. Factors related to disease-free survival (DFS) and overall survival (OS) were analyzed using multivariate Cox regression analysis.

**Results:**

In 137 r/r B-ALL patients, the 2-year cumulative incidence of recurrence (CIR), OS and DFS in patients receiving allo-HSCT after CAR-T therapy was 31.5%, 71.4%, and 60.5%, respectively. The 2-year OS and DFS in MRD-negative patients were 80.9% and 69.3%, respectively. Univariate Cox analysis showed that pretransplant MRD positivity, fungal infection, germline *EP300* mutation and somatic *TP53* mutation were associated with a poor prognosis after transplantation; a TBI-based regimen was a protective factor for survival and recurrence after transplantation. Multivariate Cox regression analysis showed that the TBI-based regimen was an independent protective factor for DFS, fungal infection and MRD positivity were independent risk factors for DFS, and tumor somatic *TP53* mutation and germline *EP300* mutation were independent risk factors for DFS and OS.

**Conclusion:**

Germline *EP300* mutation and tumor somatic *TP53* mutation are poor prognostic factors for posttransplant recurrence and survival in r/r B-ALL patients achieving CR after CAR-T therapy. The prognostic risk factors should be considered in adjusting treatment strategies to improve the efficacy of clinical diagnosis and treatment.

## Introduction

Patients with refractory/relapsed acute B-lymphocytic leukemia (r/r B-ALL) usually have a poor prognosis even if they receive chemotherapy and salvage allogeneic hematopoietic stem cell transplantation (allo-HSCT). The 1-year disease-free survival (DFS) in patients with r/r B-ALL after salvage allo-HSCT is 20%-30%, and the 5-year overall survival (OS) is approximately 30% and decreases to 10% in patients with a second recurrence (1). In recent years, chimeric antigen receptor T-cell (CAR-T) therapy, as a new immunotherapy method, has increased the complete response (CR) rate of r/r B-ALL to 70%-93% (2–5); however, 50%-67.9% of patients experience recurrence within one year (6, 7). Our previous study showed that CD19/CD22 CAR-T therapy for r/r B-ALL followed by allo-HSCT significantly improved patient survival, with a 1-year OS of 87.7% and 1-year event-free survival (EFS) of 73% (8).

Although allo-HSCT after CAR-T therapy brings more survival benefits to patients with r/r B-ALL, some patients still have a recurrence within one year after transplantation. A clinical study showed that patients with minimal residual disease (MRD) positivity (MRD+) before transplantation had a significantly lower DFS than MRD-negative (MRD-) patients at 2-years after transplantation (27.6% *vs.* 76.1%) (9).

In addition to the impact of MRD on the prognosis of transplantation, an increasing number of studies have shown that predisposition gene mutations and somatic gene mutations in tumors play a vital role in the occurrence and development of hematological malignancies. The germline predisposition of myeloid tumors was taken into account in classifying myeloid tumors and acute leukemia by the WHO in 2016 (10). Recently, researchers have found that in patients with r/r B-ALL who received CD19 CAR-T therapy, *TP53* mutation (*TP53*-mut) in somatic cells was significantly associated with a poor prognosis after CAR-T therapy (11). The 6-month survival in patients with *TP53*-mut was 51.9%, and the leukemia-free survival was only 42.4%, which was significantly lower than that in patients with *TP53* wild-type (*TP53*-wt) (11). The abovementioned data indicate the value of gene mutation in predicting the prognosis of patients receiving CAR-T therapy.

This study analyzed the prognostic factors of patients with r/r B-ALL who received allo-HSCT after achieving CR by CAR-T therapy. We systematically explored germline predisposition gene and somatic gene variation profiles and their effect on the prognosis of patients after transplantation to provide a reference for clarifying the mechanism of r/r B-ALL and developing treatment strategies to improve patient survival.

## Methods

### Patients

**Figure 1:**
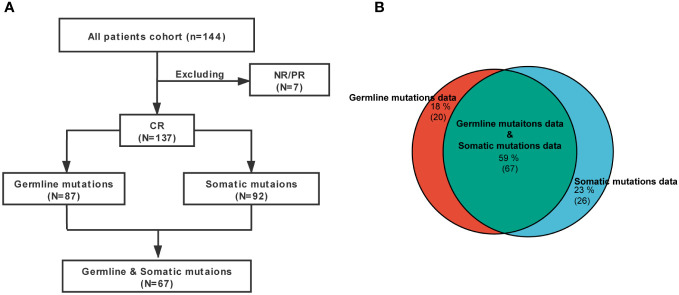
Flow chart. **(A)** Patient enrollment and sample collection flow chart. **(B)** Venn diagrams showed relationships between germline mutations data and somatic mutations data.

In total, 144 consecutive r/r B-ALL patients who underwent allo-HSCT after CD19/CD22 CAR-T therapy in our hospital from June 2017 to June 2021 were enrolled in this study, and 7 patients who did not achieve CR after CAR-T therapy were excluded from the subsequent analyses. A long-term follow-up and statistical analysis were performed on 137 r/r B-ALL patients who achieved CR after CD19/CD22 CAR-T therapy and underwent allo-HSCT (**Figure 1**), including 133 patients in our hospital (n=133) and 11 patients in the Seventh Medical Center of PLA General Hospital (n=11) (allo-HSCT was performed under the same protocol). All subjects or their guardians signed an informed consent form. This study was approved by the Medical Ethics Committees of Beijing Boren Hospital and the Seventh Medical Center of PLA General Hospital, in accordance with the Declaration of Helsinki.

### CAR-T therapy

The data were obtained from clinical trials on immunotherapy conducted in Beijing Boren Hospital (registered at www.chictr.org #ChiCTR-OIC-17013623, #ChiCTR-OIC-17013523). Lentiviruses (provided by Shanghai Yake Biotechnology Co., Ltd.) were used to construct CD19- or CD22-directed chimeric antigen receptors (CARs) with a 4-1BB costimulatory domain and CD3f signaling domain. Activation, transduction and expansion of CAR-T cells were performed in the Cell Processing Laboratory of Beijing Boren Hospital. All patients underwent lymphocyte-depleting chemotherapy with fludarabine (30 ^mg/m2^ per day, Days 5 to 3) and cyclophosphamide (250 ^mg/m2^ per day, Days 5 to 3) and then received CD19/CD22 CAR-T infusion. Then, CAR-T-cell targets were selected according to the fluorescent intensity of antigen expression of leukemia cells through flow cytometry. In total, 102 patients received CD19 CAR-T cells, 17 received CD22 CAR-T cells, and 18 patients received CD19 CART and CD22 CART twice before transplantation. Because they did not get remission after receiving CART treatment for the first time, so they had to do CART for the second time to achieve remission.

### Detection of germline predisposition gene and somatic gene mutations

Before transplantation, the peripheral blood mononuclear cells (PBMCs) of patients were collected to detect predisposition gene mutations related to hematologic malignancies or immune system diseases. The sample of bone marrow (BM) of patients were collected to analysis somatic gene mutations related to hematologic malignancies. Genomes were extracted from PBMCs and BM samples of patients before CAR-T therapy using the TIANamp blood DNA kit (Tiangen Biotechnology Co.). The extraction were performed according to the manufacturer’s instructions. The extracted DNA concentration was determined using a Nanodrop 2000 (Thermo Fisher). The genomic DNA library was constructed using KAPA HyperPlus Kits (Roche). Library concentration was measured using the Qubit 4.0 Fluorometer (Thermo Fisher). The constructed DNA library was captured using the NimbleGen SeqCap EZ Library SR (Roche) kit, and the probes contained the exon regions of 339 genes relevant to hematologic malignancies. The captured library was sequenced using the NextSeq550 (Illumina) sequencer, and the sequencing was performed using the PE150 program according to the manufacturer’s instructions. For the somatic gene mutations average sequencing depth of gene target regions was 3000×. For WES average sequencing depth was 100×.

### Bioinformatics analysis

Germline variant calling was performed with the Broad Institute’s Genome Analysis Toolkit (GATK). The variant list was further filtered to select variants of approximately 700 genes that were associated with hematological tumors and immunodeficiency. Germline variants with an allele frequency >15% in our samples and with a predicted moderate or high impact on protein function were further filtered to select variants according to their frequency in the genomAD, ExAC and 1000G databases (minor allele frequency (MAF) < 5%). Integrative Genomics Viewer (IGV) software was used to manually inspect sequencing results. The candidate genetic risk variants were validated in family members of patients by Sanger sequencing. And the interpretation of variants was based on the HGMD and ACMG guideline (12, 13).

Somatic variant calling was performed with the Broad Institute’s Genome Analysis Toolkit (GATK) and Vardict. Somatic variants were filtered to select rare variants according to their frequency in the genomAD, ExAC, and 1000G databases (MAF< 1%) and with an allele frequency >5%, but if the variant was a hotspot and the allele frequency was >1%, Integrative Genomics Viewer (IGV) software was used for the manual inspection of sequencing results.

### Hematopoietic stem cell transplantation

Myeloablative reduced toxicity regimens (RTC) were used, including total body irradiation (TBI)/FLU-based or busulfan (BU)/FLU-based regimens. Dosages were as follows: fractionated TBI (400 cGy, -9 days, 300 cGy, -8 to -7 days), FLU (30 mg/m^2^ per day, iv, -6 to -2 days), semustine (250 mg/m^2^, PO, -3 days). The patients who were first transplanted with the TBI regimen were treated with BU for the second transplantation (3.2 mg/kg per day, iv, 3-4 days for adults). Children younger than 4 years of age were treated with BU, and their dose was adjusted according to weight (14). Rabbit anti-T-lymphocyte immunoglobulin (ATG-F, 100 mg/5 ml, Astellas Pharma Inc., Tokyo, Japan, 5 mg/kg per day, iv, -5 to -2 days) was used for unrelated and haploidentical transplantation. Donor types included related haploidentical (n=104, 76.6%), unrelated (n=27, 19.0%) and sibling-identical types (n=6, 4.4%). Granulocyte colony-stimulating factor-mobilized BM combined with peripheral blood stem cells (PBSCs) was used as the graft for haploidentical transplantation. Only PBSC was used as the graft for sibling-identical or unrelated transplantation.

Cyclosporine, mycophenolate mofetil (MMF), and short-course methotrexate (MTX) were used for GVHD prophylaxis. Intravenous injection of cyclosporine (1.25 mg/kg, Q12 h) started on day -9, and oral administration was given one month later if no diarrhea occurred. MTX (15 mg/m^2^) was administered intravenously on Day 1 and then 10 mg/m^2^ on Days 3, 6 and 11. MMF (7.5 mg/kg twice per day) was given on day -2, was tapered upon neutrophil engraftment and stopped on Day 30. Acute graft-versus-host disease (aGVHD) was evaluated using the Glucksberg scale modified by Przepiorka et al. (1995), and grades III and IV should be regarded as severe aGVHD (15). Chronic graft-versus-host disease (cGVHD) was assessed using the consensus criteria by the U.S. National Institutes of Health (16).

Prophylaxis against fungi, herpesviruses, and pneumocystis pneumonia (PCP) was routinely given. Echinocandins or triazoles were used for primary or secondary prevention of fungal infections based on pretransplant medical history. Acyclovir and trimethoprim-sulfamethoxazole were used to prevent herpesvirus infection and PCP. Plasma cytomegalovirus (CMV) DNA and Epstein–Barr virus (EBV) DNA were routinely monitored by PCR 1-2 times a week after transplantation. If urinary irritation symptoms occurred, urine BKV and JCV were detected by PCR.

### Efficacy assessment

Complete response (CR), CRi, and no response (NR) are defined according to the U.S. National Comprehensive Cancer Network (NCCN) guidelines (17). According to NCCN guidelines, recurrence is defined as reappearance of blasts in the BM or peripheral blood (>5%) or any extramedullary diseases (EMDs) after CR. Refractory disease was defined as failure after 2 standard treatment regimens (18). EMDs were assessed using imaging techniques, including computed tomography (CT), positron emission tomography-computed tomography (PET-CT), and magnetic resonance imaging (MRI), on Days 15 and 30 after CAR-T infusion and 2 months after HSCT. Afterward, the patient was evaluated every 1-2 months until CR was observed. A negative status for MRD was defined as less than 0.01% BM blasts as determined by multiparameter flow cytometry (FCM, FACSCalibur, BD, USA) and/or less than 0.01% frequency of reverse transcription (RT)-PCR if the patients had leukemia-specific fusion genes or mutations. OS was defined as the interval from Day 0 (the day of transplantation) to the date of death or the last follow-up. DFS was defined as the interval from Day 0 (the day of transplantation) to the date of recurrence, death, or the last follow-up. All patients were followed up until December 31, 2021.

### Statistical analysis

The Kaplan–Meier method was used to calculate DFS and OS, the cumulative incidence of recurrence (CIR), DFS, and OS at corresponding time points (one-year, two-year). The difference in survival curves was assessed using the log-rank test. Treatment-related mortality (TRM) and CIR were assessed using a competing risk model. Recurrence mortality was defined as a competing risk of TRM. Death without recurrence was defined as a competing risk of CIR. Hazard ratios (HRs) and their 95% confidence intervals were estimated by Cox proportional hazards models. Factors with a significance of *P*< 0.1 in the univariate Cox regression analysis were included in the multivariate Cox regression model. For clinical characteristics, measurement data were compared by *t* test, and categorical data were compared by chi-square test or Fisher’s exact test. The significance level α was set as 0.05 for both sides. Statistical analysis was performed using IBM SPSS statistics 25 and R v3.6.2.

## Results

### Clinical characteristics of patients before transplantation

All patients were r/r B-ALL and heavily treated. Their clinical characteristics before transplantation are shown in **Table 1** and **Figure 1A**. The median age was 9.7 years (range: <1-56 years), and the median duration of follow-up was 22.5 months (95% CI: 20.2-24.8 months). At enrollment, 95 patients (66.0%) had recurrence, and 49 (34.0%) had refractory diseases. A total of 144 patients received CAR-T therapy, among them 137 patients achieved CR after CAR-T therapy, and 7 patients who did not achieve CR were excluded from the subsequent analysis.

**Table 1:** Characteristics of the patients.

Factor	Level	N=137 (%)	
**Sex**	Female	58	(42%)
	Male	79	(58%)
**Age**	<=18	100	(73%)
	>18	37	(27%)
**Poor cyto/mol abn**	*TP53* mutation	27	(20%)
	Ph+ *(BCR-ABL1)*	15	(11%)
	*E2A-PBX1 (TCF3-PBX1)*	8	(6%)
	*TEL-AML1 (ETV6-RUNX1)*	8	(6%)
	*MLL* rearrangement *(eg. MLL-AF4 (KMT2A-AFF1))*	5	(4%)
	*E2A-HLF (ETV6-HLF)*	2	(2%)
	Complex karyotype	9	(6%)
	Hypodiploid	1	(1%)
**EMDs**	EMDs without CNS invasion	10	(7%)
	EMDs with CNS invasion	13	(9%)
	No	114	(83%)
**CAR T-cell targets**	CD19	102	(74%)
	CD22	17	(12%)
	CD19/CD22	18	(13%)
**MRD before HSCT**	MRD-	101	(74%)
	MRD+	36	(26%)
**Conditioning regimen**	BU-based	53	(39%)
	TBI-based	84	(61%)
**Donor type**	Haplo	104	(76%)
	Unrelated	27	(20%)
	Identical sibling	6	(4%)
**Blood type match**	No	65	(47%)
	Yes	72	(53%)

Among the 137 patients with CR after CAR-T therapy, 23 (16.8%) had EMDs of whom 13 had CNSL (9.5%). Eight patients (5.8%) had a second transplantation. Before transplantation, 101 patients (73.7%) were MRD negative, and 36 patients (26.3%) were MRD positive (7 patients who did not achieve CR after CAR-T therapy were not assessed for MRD). The median time interval from CAR-T-cell infusion to allo-HSCT was 51 days (range: 34-94 days). The median number of infused mononuclear cells (MNCs) was 9×10^8^/kg (3.30-45.44×10^8/kg), and the median number of CD34+ cells was 5.39×10^6^/kg (0.65-27.98×10^6^/kg).

### Transplantation, graft-versus-host disease and infection

Durable complete chimerism was achieved in all but one recipient. One patient had primary transplantation failure but achieved durable and stable hematopoietic reconstruction after the second transplantation. The median time to neutrophil and platelet engraftment was 15 days (9-21 days) and 13 days (5-33 days). The proportions of patients with grade II-IV and severe aGVHD were 27% and 14.5%, respectively. Twenty-five patients (18.2%) developed cGVHD (18 localized and 7 extensive). The incidence of CMV and EBV activation was 20.1% and 12.5%, respectively. There were 32 cases (22.2%) of hemorrhagic cystitis, and the incidence of viral disease was 20.4% (4 cases of CMV pneumonia, 1 case of PTLD, and 23 cases of viral cystitis). Bacterial infection occurred in 22 cases (15.3%), and fungal infection occurred in 5 cases (3.5%).

### Survival, recurrence, and transplant-related death

**Figure 2:**
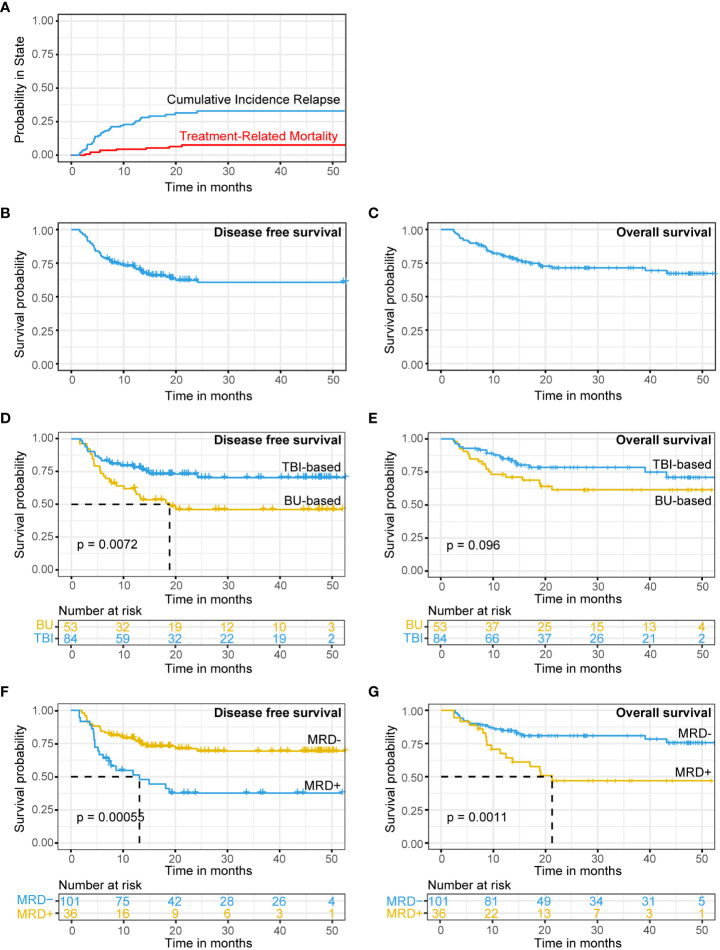
CIR, TRM, DFS and OS in r/r B-ALL patients treated with CAR-T therapy followed by allo-HSCT. **(A–C)** CIR **(A)**, TRM **(A)**, DFS **(B)** and OS **(C)** for patients with r/r B-ALL. **(D, E)** DFS **(D)** and OS **(E)** analysis for patients with TBI-based or BU-based conditioning regimens. **(F, G)** DFS **(F)** and OS **(G)** analysis for patients with MRD-positive or MRD-negative before transplantation. CIR, TRM, DFS and OS analysis using Kaplan-Meier curves.

By December 31, 2021, 100 out of 137 r/r B-ALL patients had survived. The median follow-up time was 27.9 months (95% CI: 23.55-32.25 months). In patients who received transplantation after achieving CR by CAR-T therapy, the 1-year and 2-year CIR 23.7% (95% CI: 15.4-29.3%) and 31.5% (95% CI: 25.3-42.7%; **Figure 2A**), respectively. The 1-year and 2-year OS rates were 80.4% (95% CI: 72.6-86.2%) and 71.4% (95% CI: 62.3-78.7%; **Figure 2C**), respectively. The 1-year and 2-year DFS rates were 71.0% (95% CI: 62.5-77.9%) and 60.5% (95% CI: 50.9-68.8%; **Figure 2B**), respectively.

Forty-one (29.9%) patients had recurrence after transplantation. The recurrence rate was 23.8% (24/101) in MRD-negative patients and 47.2% (17/36) in MRD-positive patients. Thirty-seven patients (27.0%) died (28 died of recurrence, 2 died of GVHD, 4 died of infection, 2 died of multiorgan failure, and 1 died of thrombotic microangiopathy). The overall TRM was 6.5% (**Figure 2A**).

### Relationship between clinical parameters and disease-free survival and overall survival after transplantation

First, we investigated the relationship between clinical parameters (patient sex, age, *BCR-ABL1* (Ph+), *E2A-PBX1*, *E2A-HLF*, *TEL-AML1*, complex karyotypes, EMDs, conditioning regimens, transplant-related parameters, grafts, viral activation, and graft-versus-host disease) and DFS and OS of patients after transplantation.

Univariate Cox regression analysis showed that patients receiving the TBI-based regimen had a significantly higher DFS than patients receiving the BU-based regimen (DFS: TBI-based *vs.* BU-based, HR = 0.47, 95% CI: 0.27-0.83, p -value = 0.0087; OS: TBI-based *vs.* BU-based, HR = 0.58, 95% CI: 0.31–1.1, p value = 0.1; **Table 2**; **Figures 2D, E**).

**Table 2:** Univariate cox regression analysis.

	DFS		OS	
	HR (95% CI for HR)	P value	HR (95% CI for HR)	P value
**Sex**	0.88 (0.5-1.5)	0.65	1 (0.53-2)	0.96
**Age**	1 (0.98-1)	0.8	1 (0.98-1)	0.5
**Ph+ (*BCR-ABL1*)**	1.1 (0.46-2.5)	0.88	0.96 (0.34-2.7)	0.93
***E2A-PBX1***	1.1 (0.34-3.5)	0.88	0.87 (0.21-3.6)	0.85
***E2A-HLF***	1.5 (0.2-11)	0.69	1.5 (0.21-11)	0.69
***TEL-AML1***	1.1 (0.33-3.4)	0.92	1 (0.24-4.2)	0.98
**Complex karyotype**	0.27 (0.038-2)	0.2	0.41 (0.056-3)	0.37
**EMDs**	1.4 (0.69-2.8)	0.36	1.2 (0.53-2.7)	0.67
**MRD(+) before HSCT**	2.6 (1.5-4.6)	0.00088	2.8 (1.5-5.4)	0.0017
**Conditioning regimen - TBI**	0.47 (0.27-0.83)	0.0087	0.58 (0.31-1.1)	0.1
**Haplo**	1.1 (0.55-2)	0.87	1.5 (0.64-3.3)	0.37
**Unrelated**	0.97 (0.48-1.9)	0.93	0.6 (0.23-1.5)	0.29
**Identical sibling**	0.89 (0.22-3.7)	0.88	1.1 (0.27-4.7)	0.86
**Blood type match**	1.1 (0.65-2)	0.63	1.5 (0.77-2.9)	0.24
**WBC engraftment time**	0.97 (0.87-1.1)	0.51	0.93 (0.82-1)	0.23
**Platelet’s engraftment time**	0.99 (0.93-1.1)	0.87	0.96 (0.88-1)	0.3
**aGVHD**	0.9 (0.46-1.8)	0.75	1.2 (0.59-2.5)	0.59
**Sever aGVHD**	1.1 (0.48-2.4)	0.86	1.6 (0.7-3.6)	0.26
**cGVHD**	0.58 (0.25-1.4)	0.21	0.53 (0.19-1.5)	0.23
**Extensive cGVHD**	0.35 (0.049-2.6)	0.3	3.8e-08 (0-Inf)	1
**Bacterial infection**	0.47 (0.17-1.3)	0.15	0.51 (0.16-1.7)	0.27
**Fungal infection**	12 (4.1-37)	0.000009	6.6 (2-22)	0.002
**Viraemia**	1.5 (0.82-2.6)	0.2	1.2 (0.59-2.3)	0.66
**Viral disease**	1.5 (0.36-6.2)	0.57	2.8 (0.67-12)	0.16
**Viral cystitis**	0.86 (0.43-1.7)	0.68	1.1 (0.53-2.4)	0.74

In addition, we found that fungal infection significantly increased the risk of DFS and OS (DFS: fungal infection *vs.* no fungal infection, HR = 12, 95% CI: 4.1-37, p value < 0.0001; OS: fungal infection *vs.* no fungal infection, HR = 6.6, 95% CI: 2-22, p value = 0.002; **Table 2**).

### Relationship between pretransplant MRD and the prognosis of patients receiving CAR-T therapy followed by transplantation

Univariate Cox analysis showed that pretransplant MRD was associated with DFS and OS (**Table 2**). Pretransplant MRD-positive patients had a significantly higher risk of recurrence or death than MRD-negative patients (DFS: MRD-positive *vs.* MRD-negative: HR = 2.6, 95% CI: 1.5-4.6, p value < 0.0001; OS: MRD-positive *vs.* MRD-negative: HR = 2.8, 95% CI: 1.5-5.4, p value = 0.0017; **Table 2**; **Figures 2F, G**). The 1-year recurrence rate was 45.5% (95% CI: 24.9-60.5%) in MRD-positive patients, which was significantly higher than the 20.8% (95% CI: 12.2-28.6%) in MRD-negative patients (p = 0.017). Similarly, the 1-year DFS and OS in MRD-positive patients were 47.9% and 67.4%, significantly lower than 77.8% and 84.8% in MRD-negative patients, respectively (p=0.005, 0.059). The 2-year DFS and OS rates in MRD-negative patients in our cohort were 69.3% and 80.9%, respectively.

Moreover, in MRD-positive patients, our analysis showed that BU-based conditioning regimen was associated with poor OS and DFS when compared with TBI-based regimen (MRD-positive & BU-based: mOS=18.9 months, MRD-positive & TBI-based: mOS =not reached, p value = 0.055, **Figure S2F**; MRD-positive & BU-based: mDFS=7.67 months, MRD-positive & TBI-based: mDFS=not reached, p value = 0.085, **Figures S1A, B**). However, there was no significant difference in posttransplant DFS and OS in MRD-negative patients between the BU-based and TBI-based conditioning regimens (p value > 0.05, **Figures S1A, B**).

### Relationship between germline predisposition genes of hematologic malignancies and somatic gene variation and posttransplant prognosis

In all 137 patients in this cohort, 87 patients were evaluated for germline predisposition variants, and 92 patients were evaluated for somatic gene variants. Sixty-seven patients underwent both germline predisposition gene and somatic gene variation sequencing (**Figure 1B**). The inclusion and exclusion criteria and the status of molecular testing are shown in **Figures 1A, B**.

**Figure 3:**
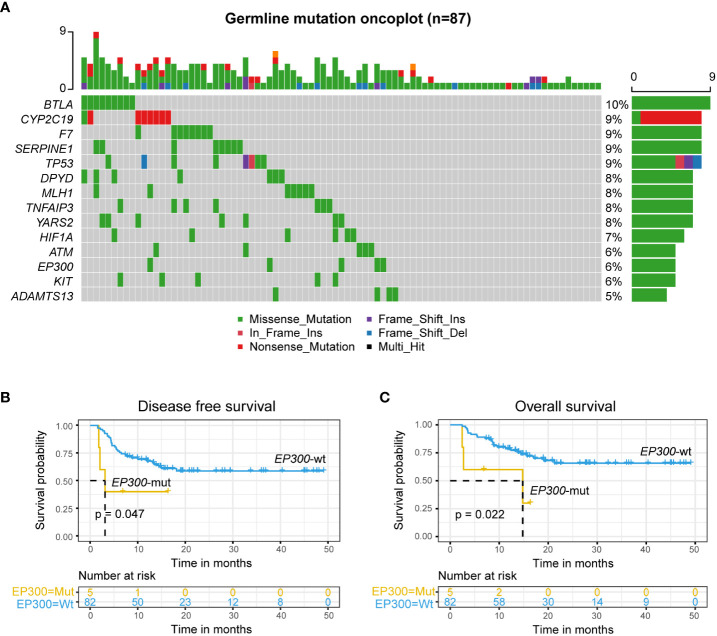
Germline *EP300* variation associated with poor prognosis. **(A)** Germline predisposition genes variation of leukemia. **(B, C)** DFS **(B)** and OS **(C)** analysis for patients with mutated or wild-type *EP300* in r/r B-ALL patients treated with CAR-T therapy followed by allo-HSCT using Kaplan-Meier curves.

The analysis of germline predisposition gene variants showed that genes with the highest mutation frequency were as follows: *BLTA, F7, KIT, SERPINE1, TP53, DPYD, MLH1, TNFAIP3, YARS2, HIFIA, ATM, EP300*, and *ADAMTS13* (**Figure 3A**). Univariate Cox regression analysis of DFS and OS on most frequency mutant genes (**Table S1**) showed that *EP300*-mut patients had a significantly lower DFS and OS than *EP300*-wt patients (*EP300*-mut: mDFS=3.13 months; *EP300*-wt: mOS=not reached, log-rank p value = 0.047; *EP300*-mut: mOS=14.8 months; *EP300*-wt: mOS=not reached, log-rank p value = 0.022; **Figures 3A, B**), and *EP300*-mut patients presented a significantly increased proportion of EMDs (*EP300*-mut *vs. EP300*-wt: 60% *vs.* 12.2%, p value = 0.024).

**Figure 4:**
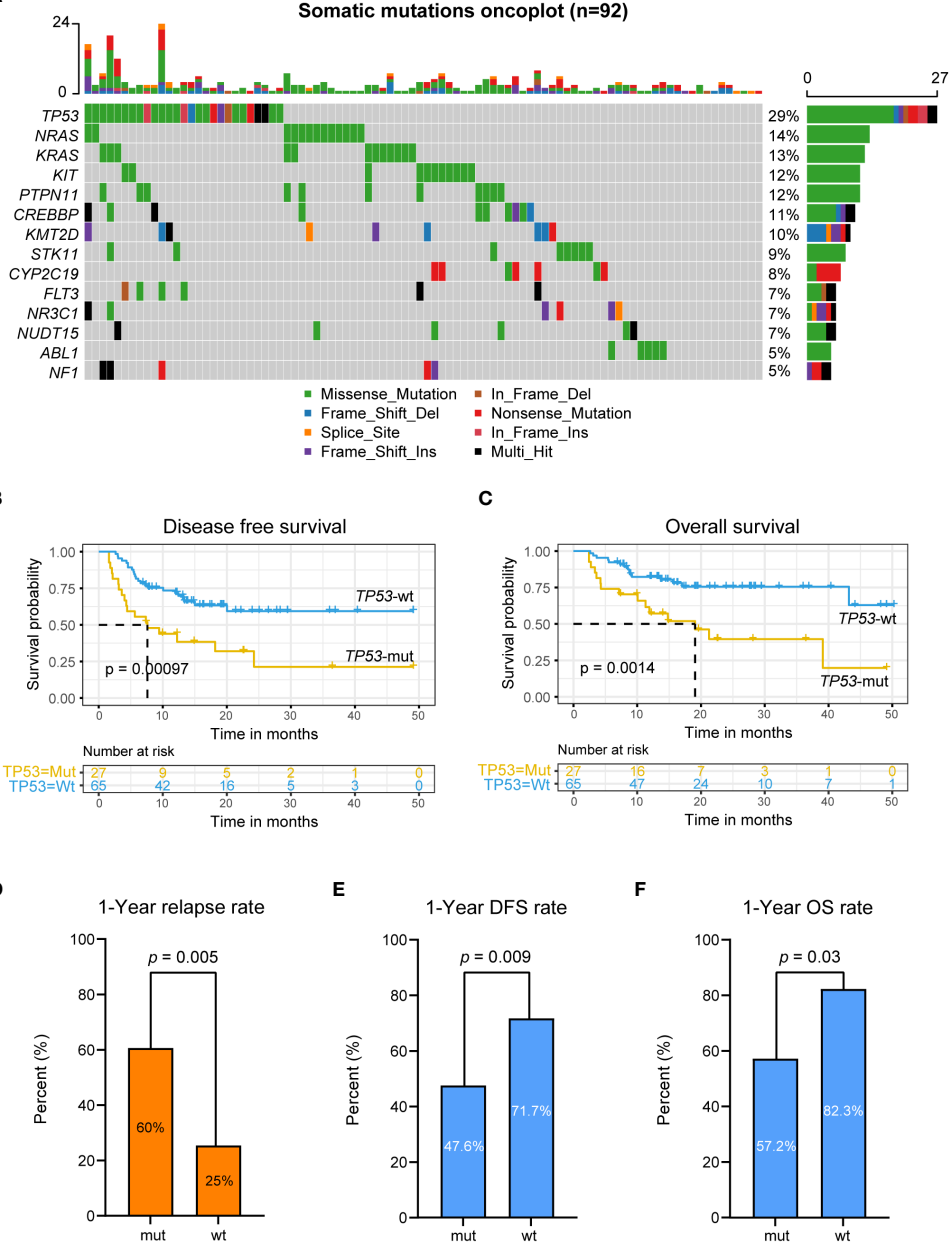
Somatic *TP53* variation associated with poor prognosis. **(A)** Somatic genes variation oncoplot. **(B, C)** DFS **(B)** and OS **(C)** analysis for patients with mutated or wild-type *TP53* in r/r B-ALL patients treated with CAR-T therapy followed by allo-HSCT using Kaplan-Meier curves. **(D–F)** 1-year CIR, DFS rate and OS rate for patients with mutated or wild-type *TP53*.

We also analyzed gene mutations in tumor somatic cells in 92 patients. The results showed that genes with a high mutation frequency were *TP53, NRAS, KRAS, PTPN11, CREBBP, KMT2D, STK11, FLT3, NR3C1, ABL1*, and *NF1* (**Figure 4A**). Univariate Cox regression analysis of DFS and OS on genes with most frequency mutant genes (**Table S2**) showed that somatic *TP53* mutations were a prognostic risk factor in patients who underwent allo-HSCT after achieving CR by CAR-T therapy (DFS: HR = 0.37, 95% CI: 0.2-0.68, p value = 0.0015; OS: HR = 0.33, 95% CI: 0.16-0.67, p value = 0.0024; **Table S2**). DFS and OS in *TP53*-mut patients were also significantly worse than those in *TP53*-wt patients (DFS: *TP53*-mut, mDFS=7.6 months; *TP53*-wt, mDFS=not reached, log-rank p value < 0.0001, **Figure 4B**; OS: *TP53*-mut, mOS=19.1 months; *TP53*-wt, mOS=not reached, log-rank p value = 0.0014, **Figure 4C**).

The 1-year CIR in *TP53*-mut patients was 60.0%, which was significantly higher than that in *TP53*-wt patients (*TP53*-mut *vs. TP53*-wt: 60.0% *vs.* 25.0%, p value = 0.005; **Figure 4D**). The 1-year DFS and OS in *TP53*-mut patients were 38.4% and 57.2%, respectively, significantly lower than 69.8% and 82.3% in *TP53*-wt patients (p value = 0.009, 0.03; **Figures 4E, F**). Seventeen out of 27 patients with somatic *TP53* mutation had recurrence after allo-HSCT. Fifteen patients with somatic *TP53* mutations died, including 13 relapse-related deaths and 2 non-relapse mortality.

We further performed analysis on conditioning regimens for patients with *TP53*-mut and *TP53*-wt. The results showed that for *TP53*-mut patients, TBI-based or BU-based regimens showed no significant improvement in DFS and OS (p> 0.05, **Figures S2A, B**); for *TP53*-wt patients, the TBI-based conditioning regimen significantly increased the DFS of patients (p value = 0.025; **Figure S2A**).

In conclusion, univariate Cox regression analysis of germline predisposition gene and somatic gene mutations showed that germline *EP300* mutations and somatic *TP53* mutations were associated with a high risk of recurrence and death after allo-HSCT.

### Multivariate Cox regression analysis of the relationship between clinical characteristics, gene mutation profile and prognosis

**Figure 5:**
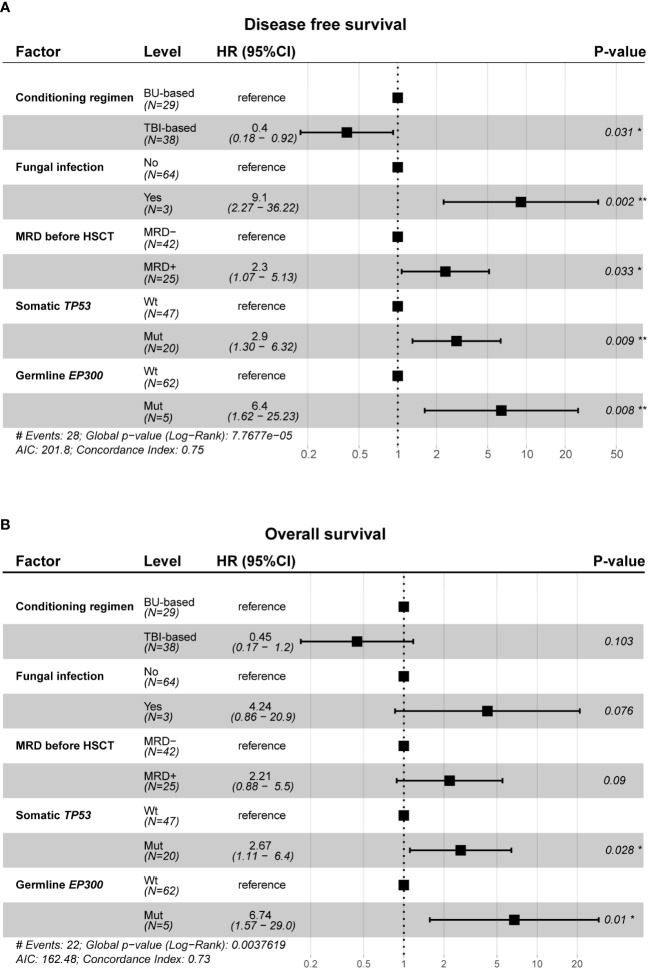
Multivariate cox regression analysis between clinical characteristics, gene mutation profile and prognosis. **(A, B)** Forest plot of hazard ratios for conditioning regimen, fungal infection, MRD before allo-HSCT, somatic TP53 mutations and germline *EP300* mutations associated with DFS **(A)** and OS **(B)**. Two-sided Wald-test p-value are reported. *p<0.05, **p<0.01.

Multivariate Cox regression analysis was used to further investigate the relationship of germline predisposition gene and somatic gene mutations with DFS and OS in patients receiving allo-HSCT after CAR-T therapy. The factors significantly related to prognosis in univariate Cox regression (clinical parameters and germline predisposition gene and somatic gene mutations) were included in multivariate analysis. The results showed that MRD before transplantation, somatic *TP53* mutations and germline *EP300* mutations were independent risk factors for DFS (DFS: global log-rank p value < 0.0001, C-index = 0.75, MRD+: HR = 2.3, p -value = 0.033; somatic *TP53*-mut: HR = 2.9, p value = 0.009; germline *EP300*-mut: HR = 6.4, p value = 0.008; **Figure 5A**). Somatic *TP53* mutations and germline *EP300* mutations were independent risk factors for OS (OS: global log-rank p value = 0.0038, C-index = 0.73, somatic *TP53*-mut: HR = 2.67, p value = 0.028; germline *EP300*-mut: HR = 6.74, p value = 0.01; **Figure 5B**).

## Discussion

Patients with r/r B-ALL usually have a poor prognosis. CAR-T therapy, as a novel immunotherapy in recent years, has dramatically increased the CR rates of such patients (19–21); however, more than 50% of CR patients who only received CAR-T therapy relapse within one year (6, 19, 20, 22, 23). CAR-T therapy followed by allo-HSCT may be a valuable treatment strategy to further improve the prognosis of patients with r/r B-ALL. This study reported the follow-up results of patients with r/r B-ALL who received allo-HSCT after CR by CAR-T therapy in our hospital since 2017 and analyzed the characteristics that affected the prognosis of patients after transplantation.

In our cohort, the 2-year CIR, DFS and OS in patients who received allo-HSCT after CR by CAR-T therapy were 31.5%, 60.5% and 71.4%, respectively, similar to previous studies in China and other centers in other countries (8, 9, 11, 24, 25). Moreover, the recurrence rate and mortality rate were lower than those in patients who only received CAR-T therapy reported in the literature, indicating that the strategy of allo-HSCT after CAR-T therapy can improve the prognosis of patients with r/r B-ALL. In addition, compared with allo-HSCT patients who had not received CAR-T therapy, our patients showed no increase in transplant-related toxicity, consistent with the literature results (9). Our study showed that conditioning regimens and posttransplant fungal infection were associated with a poor prognosis. The patients receiving the BU-based regimen had a lower DFS than the patients receiving the TBI-based regimen, consistent with previous studies in ALL patients (26, 27), suggesting that the TBI-based conditioning regimen should be the first choice for allo-HSCT in r/r B-ALL patients. In the stratified analysis, TBI-based regimens improved DFS and OS in MRD-positive patients. At the same time, there was no significant difference in recurrence and survival of MRD-negative patients regardless of which regimen was used. For patients with somatic *TP53* mutations, the TBI-based regimen did not improve their prognosis compared with the BU-based regimen; for patients with wild-type *TP53*, the DFS of patients who received the TBI-based regimen was superior to that of patients receiving the BU-based regimen. In conclusion, TBI-based conditioning regimens have an overall survival advantage in patients with r/r B-ALL who undergo allo-HSCT after CR by CAR-T therapy.

There is a consensus that MRD is an independent prognostic indicator for B-ALL patients (28). Among the patients with CR after CAR-T therapy, MRD-positive patients usually have a higher risk of recurrence (29). In our study, FCM and RT-PCR were used to detect MRD. Univariate analysis showed that MRD-positive patients had significantly lower DFS and OS than MRD-negative patients. The 2-year recurrence rate, DFS and OS in MRD-negative patients in our cohort were 27.2%, 69.3% and 80.9%, respectively, consistent with previous reports (9, 11, 30). Zhao et al. (9) found that in a cohort of Chinese r/r B-ALL patients who were MRD-negative, the 2-year DFS and OS after CAR-T therapy followed by allo-HSCT were 65.6% and 77.0%, respectively, which were similar to our data. In multivariate analysis, MRD-positivity remained a significant independent risk factor for DFS. Therefore, MRD-positivity is significantly associated with a poor prognosis in r/r B-ALL patients receiving allo-HSCT after CAR-T therapy. Our study suggests that a TBI-based regimen can improve DFS and OS in MRD-positive patients.

In recent years, it has been reported that germline and tumor somatic gene variation affects the treatment response and prognosis of patients with hematological tumors (31–33). In this study, we applied NGS to systematically analysis germline predisposition gene variants and somatic gene variants in r/r B-ALL patients who presented a higher frequency of mutations and analyzed the effects of relevant germline and somatic gene variants on the prognosis of patients receiving allo-HSCT after CAR-T therapy. *EP300* p. I997 V is a deleterious inactivating mutation in patients with hematologic malignancies undergoing allo-SCT (34). Somatic *EP300* mutation is primarily seen in diffuse large B-cell lymphoma (DLBCL) and follicular lymphoma (FL), resulting in abnormal acetylation of p53 and BCL6 and thereby promoting tumorigenesis (35). Li, et al. (36) reported that the *EP300* p. I997 V inactivating mutation is an indicating factor for poor prognosis in DLBCL patients treated with R-CHOP. Germline *EP300* mutations can cause autosomal dominant Rubinstein-Taybi syndrome (RSTS) and are associated with predisposition to lymphoma (37). However, our study revealed the relationship between germline *EP300* mutations and the prognosis of r/r B-ALL patients for the first time. Germline *EP300* p. I997 V was detected in 5 patients in our cohort, an independent risk factor for DFS and OS. Those patients were prone to EMDs and a poor prognosis after allo-HSCT. This suggests that this variation should be considered in donor selection.

*TP53* is the most frequently mutated gene in human cancers. Its translation product p53 suppresses tumorigenesis through transcriptional regulation of a network of target genes that play a role in various cellular processes (38). Somatic *TP53* mutations are associated with treatment resistance and poor prognosis of different cancers, which has also been reported in patients receiving CAR-T therapy (39–43). In our study, in patients with *TP53* mutations after CAR-T therapy and allo-HSCT, the 1-year CIR was 60%, twice as high as that in wild-type patients; the 1-year DFS and OS rates were 38.4% and 57.2%, respectively, which was much lower than those in wild-type patients. Multivariate analysis also showed that *TP53* mutation was a significant independent risk factor for DFS and OS. Zhang et al. found similar result in their research study using multivariable analysis, and the existence of *TP53* mutations is related to poor OS and LFS. Bridging allogeneic HSCT after CAR T cell therapy is associated with improved OS and LFS (11). However, some studieshave shown that CD19-22 CART sequential therapy is safe and effective in r/r B-ALL (21).

With the current treatment strategy for r/r B-ALL patients, other factors (age, sex, unfavorable fusion gene and/or chromosomal abnormalities, EMDs, different donor types, different blood types of donors/patients, aGVHD and cGVHD) showed no significant effect on the prognosis of patients after transplantation.

## Conclusion

CAR-T therapy followed by allo-HSCT can improve survival in patients with r/r B-ALL. The 2-year DFS and OS in MRD-negative patients in our cohort were 69.3% and 80.9%, respectively. TBI-based conditioning regimens can significantly improve the prognosis of patients; fungal infection is significantly related to the risk of recurrence and death after transplantation. MRD positivity remains an independent risk factor for the prognosis of patients receiving CAR-T therapy and allo-HSCT. Germline *EP300* mutations and somatic *TP53* mutations are significantly associated with a poor prognosis after allo-HSCT. For patients with r/r B-ALL, it is necessary to provide an individualized and precise conditioning regimen and posttransplant maintenance therapy based on MRD status and molecular variation characteristics.

## Data availability statement

The original contributions presented in the study are included in the article/**Supplementary Material**. Further inquiries can be directed to the corresponding authors.

## Ethics statement

The studies involving human participants were reviewed and approved by the Medical Ethics Committees of Beijing Boren Hospital and the Seventh Medical Center of PLA General Hospital. The patients/participants provided their written informed consent to participate in this study.

## Author contributions

Conception and design: ZL, KY, QZ, and TW. Administrative support: XZ. Provision of study materials or patients: ZL, YS, YZ, FW, XPW, JL, XXW, XZ, and TW. Collection and assembly of data: ZL, KY, YS, YZ, FW, XPW, JL, XXW, QZ, and TW. Data analysis and interpretation: ZL, KY, QZ, and TW. Manuscript writing: All authors Final approval of manuscript: All authors Accountable for all aspects of the work: All authors. All authors contributed to the article and approved the submitted version.

## Supplementary material

Supplementary Table 1 Univariate cox regression analysis of high population mutation frequency of germline genes. (Mutation n>=5).

Supplementary Table 2 Univariate cox regression analysis of high population mutation frequency of somatic genes. (Mutation n>=5).

Supplementary Figure 1 Survival analysis of conditioning regimen and MRD status before allo-HSCT.

Supplementary Figure 2 Survival analysis of somatic *TP53* mutation status and conditioning regimen.

Supplementary Figure 3 Multi-cox regression in each MRD before allo-HSCT subgroup. **(A, B)** Forest plot of hazard ratios for conditioning regimen, fungal infection, somatic *TP53* mutations and germline *EP300* mutations associated with DFS **(A)** and OS **(B)**. Two-sided Wald-test p-value is reported.
