# Supplementary material for: CAR-T therapy followed by allogeneic hematopoietic stem cell transplantation for refractory/relapsed acute B lymphocytic leukemia: Long-term follow-up results
Source: Front Oncol. 2023 Jan 4;12:1048296. doi: 10.3389/fonc.2022.1048296 (PMC9846489; doi:10.3389/fonc.2022.1048296)
Supplement: Supplementary Table 1 — Univariate cox regression analysis of high population mutation frequency of germline genes. (Mutation n>=5). [file Table_1.docx]

**Supplemental Table 1.** Univariate cox regression analysis of high population mutation frequency of germline genes. (Mutation n>=5)

|  |  | | DFS | | | OS | |
| --- | --- | --- | --- | --- | --- | --- | --- |
|  | level | HR  (95% CI for HR) | | p-value | HR  (95% CI for HR) | | p-value |
| *BTLA* | Wt | 1  (0.35-2.9) | | 0.98 | 0.96  (0.29-3.2) | | 0.94 |
| *CYP2C19* | Wt | 1.2  (0.38-4.1) | | 0.73 | 1.4  (0.34-6.1) | | 0.63 |
| *KIT* | Wt | 0.53  (0.21-1.4) | | 0.19 | 0.83  (0.25-2.8) | | 0.76 |
| *F7* | Wt | 1.6  (0.38-6.7) | | 0.52 | 1  (0.24-4.2) | | 1 |
| *SERPINE1* | Wt | 7.9e+07  (0-Inf) | | 1 | 7.7e+07  (0-Inf) | | 1 |
| *TP53* | Wt | 0.71  (0.25-2) | | 0.52 | 0.84  (0.25-2.8) | | 0.78 |
| *DPYD* | Wt | 0.83  (0.25-2.7) | | 0.76 | 0.58  (0.17-1.9) | | 0.38 |
| *MLH1* | Wt | 0.73  (0.22-2.4) | | 0.61 | 0.55  (0.17-1.8) | | 0.34 |
| *TNFAIP3* | Wt | 1.4  (0.33-5.7) | | 0.67 | 0.81  (0.19-3.5) | | 0.77 |
| *YARS2* | Wt | 1.4  (0.34-6) | | 0.62 | 2.1  (0.29-16) | | 0.46 |
| *HIF1A* | Wt | 0.47  (0.16-1.3) | | 0.15 | 0.5  (0.15-1.7) | | 0.26 |
| *ATM* | Wt | 1  (0.24-4.3) | | 0.98 | 7.3e+07  (0-Inf) | | 1 |
| *EP300* | Wt | 0.32  (0.097-1) | | 0.06 | 0.27  (0.079-0.89) | | 0.032 |
| *ADAMTS13* | Wt | 1.5  (0.21-11) | | 0.67 | 0.86  (0.12-6.4) | | 0.88 |
