# Supplementary material for: CAR-T therapy followed by allogeneic hematopoietic stem cell transplantation for refractory/relapsed acute B lymphocytic leukemia: Long-term follow-up results
Source: Front Oncol. 2023 Jan 4;12:1048296. doi: 10.3389/fonc.2022.1048296 (PMC9846489; doi:10.3389/fonc.2022.1048296)
Supplement: Supplementary Table 2 — Univariate cox regression analysis of high population mutation frequency of somatic genes. (Mutation n>=5). [file Table_2.docx]

**Supplemental Table 2.** Univariate cox regression analysis of high population mutation frequency of somatic genes. (Mutation n>=5)

|  |  | DFS | | OS | |
| --- | --- | --- | --- | --- | --- |
| Gene | level | HR  (95% CI for HR) | p-value | HR  (95% CI for HR) | p-value |
| *TP53* | Wt | 0.37  (0.2-0.68) | 0.0015 | 0.33  (0.16-0.67) | 0.0024 |
| *NRAS* | Wt | 1.2  (0.48-3.1) | 0.69 | 0.78  (0.3-2) | 0.61 |
| *KRAS* | Wt | 0.9  (0.38-2.1) | 0.8 | 1.2  (0.41-3.4) | 0.77 |
| *PTPN11* | Wt | 1.6  (0.57-4.5) | 0.37 | 2.6  (0.6-11) | 0.2 |
| *CREBBP* | Wt | 1.3  (0.45-3.6) | 0.66 | 1.4  (0.41-4.5) | 0.61 |
| *KMT2D* | Wt | 1  (0.36-2.8) | 0.98 | 1.6  (0.39-6.8) | 0.5 |
| *STK11* | Wt | 0.74  (0.26-2.1) | 0.56 | 1.2  (0.28-4.9) | 0.83 |
| *FLT3* | Wt | 7.6e+07  (0-Inf) | 1 | 7.5e+07  (0-Inf) | 1 |
| *NR3C1* | Wt | 0.4  (0.14-1.1) | 0.1 | 0.42  (0.15-1.2) | 0.11 |
| *ABL1* | Wt | 1.3  (0.31-5.4) | 0.72 | 2.1  (0.28-15) | 0.48 |
| *NF1* | Wt | 0.45  (0.14-1.5) | 0.19 | 0.63  (0.15-2.7) | 0.53 |
