# Supplementary figures and images for: CAR-T therapy followed by allogeneic hematopoietic stem cell transplantation for refractory/relapsed acute B lymphocytic leukemia: Long-term follow-up results
Source: Front Oncol. 2023 Jan 4;12:1048296. doi: 10.3389/fonc.2022.1048296 (PMC9846489; doi:10.3389/fonc.2022.1048296)

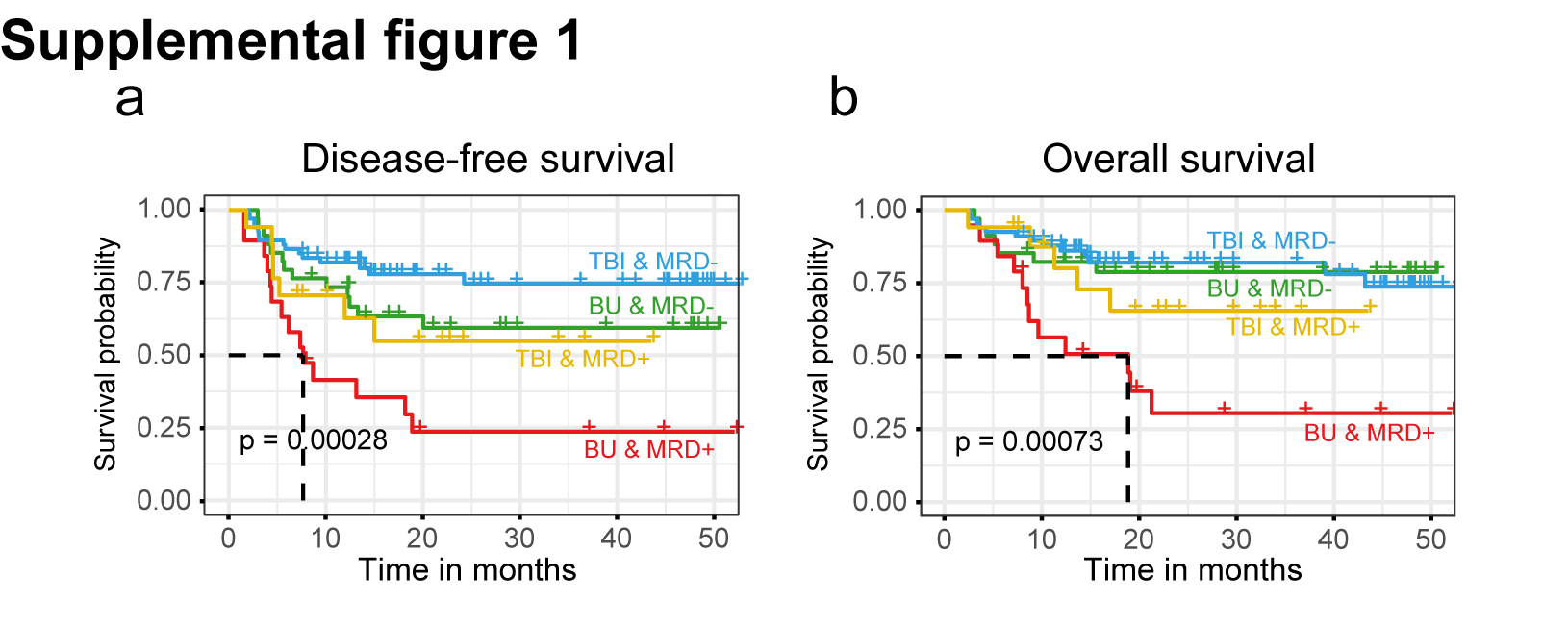

Supplement: Supplementary Figure 1 — Survival analysis of conditioning regimen and MRD status before allo-HSCT. [file Image_1.tif]

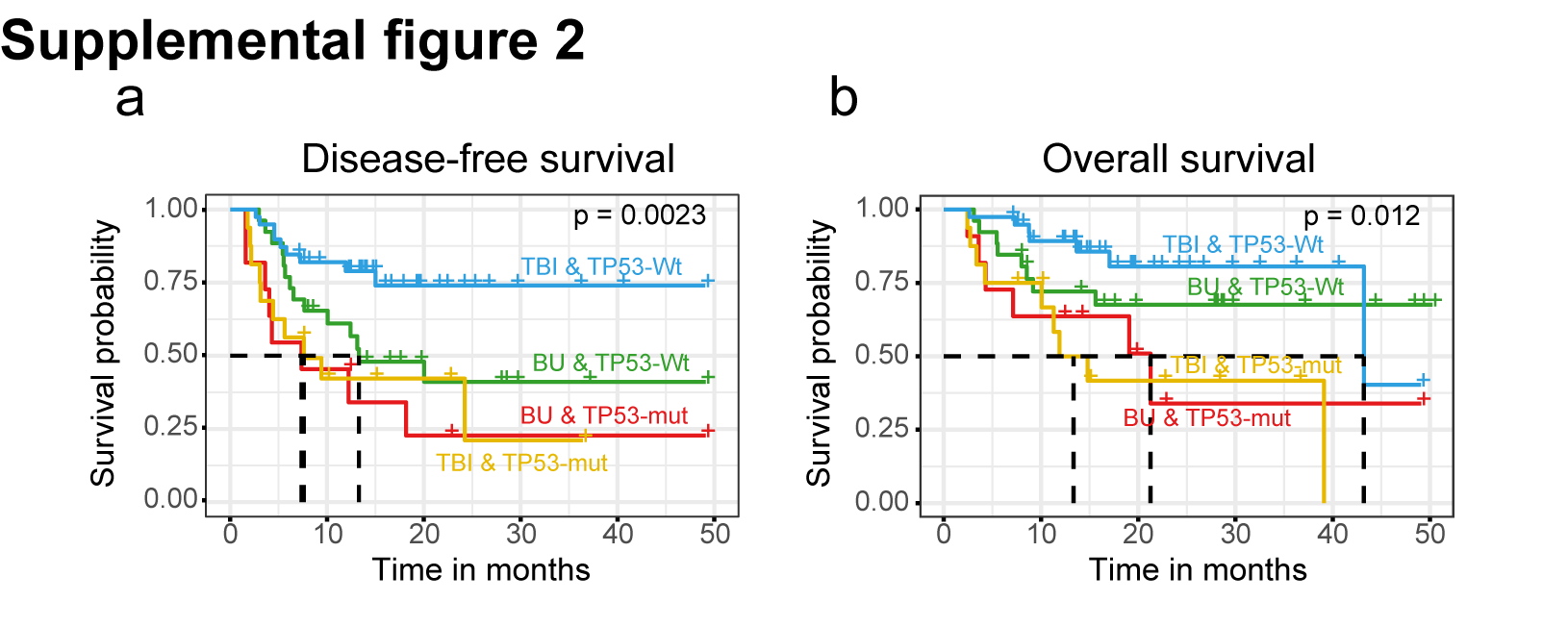

Supplement: Supplementary Figure 2 — Survival analysis of somatic TP53 mutation status and conditioning regimen. [file Image_2.tif]

A

DFS

MRD+

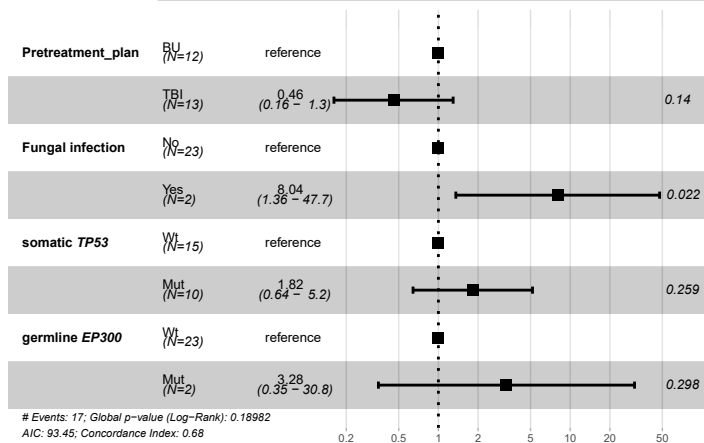

MRD-

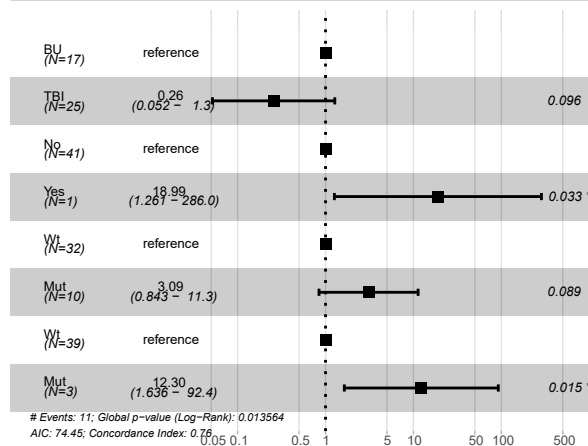

B

OS

MRD+

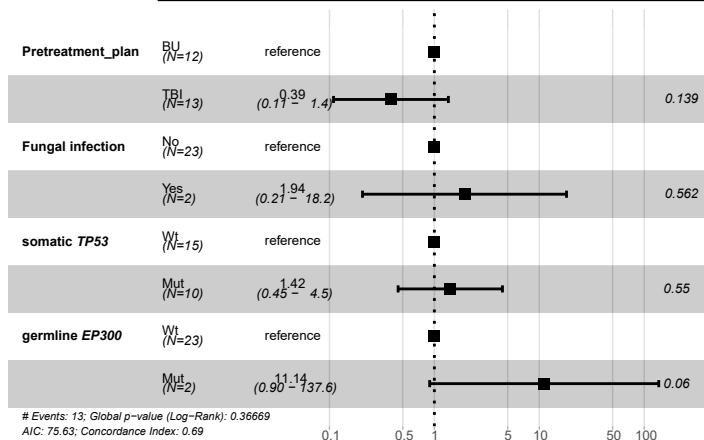

MRD-

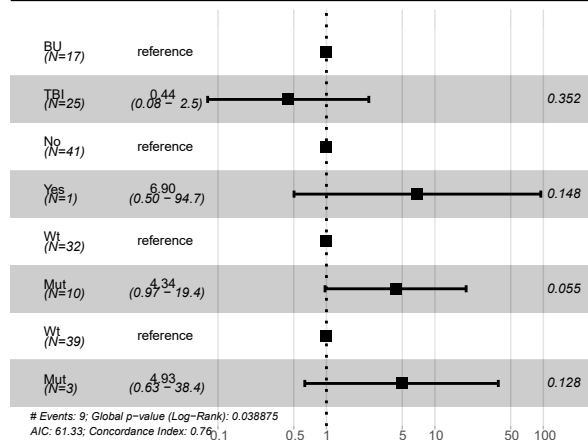

Supplement: Supplementary Figure 3 — Multi-cox regression in each MRD before allo-HSCT subgroup. (A, B) Forest plot of hazard ratios for conditioning regimen, fungal infection, somatic TP53 mutations and germline EP300 mutations associated with DFS (A) and OS (B). Two-sided Wald-test p-value is reported. [file Image_3.pdf]
